# Radiation‐Resistant Aluminum Alloy for Space Missions in the Extreme Environment of the Solar System

**DOI:** 10.1002/adma.202513450

**Published:** 2025-12-15

**Authors:** Patrick D. Willenshofer, Matheus A. Tunes, Hi T. Vo, Lukas Stemper, Markus Alfreider, Oliver Renk, Graeme Greaves, Daniel Kiener, Peter J. Uggowitzer, Stefan Pogatscher

**Affiliations:** ^1^ Chair of Non‐Ferrous Metallurgy Deparment Metallurgy Montanuniversität Leoben Franz‐Josef‐Straße 18 8700 Leoben Austria; ^2^ Materials Science and Technology Division Los Alamos National Laboratory Los Alamos NM 87545 USA; ^3^ AMAG rolling GmbH Lamprechtshausener Str. 61 5282 Ranshofen Austria; ^4^ Chair of Materials Physics Department Materials Science Montanuniversität Leoben Franz‐Josef‐Straße 18 8700 Leoben Austria; ^5^ Chair of Physical Metallurgy Department Materials Science Montanuniversität Leoben Franz‐Josef‐Straße 18 8700 Leoben Austria; ^6^ United Kingdom Atomic Energy Authority Culham Campus OX14 3DB Abingdon UK

**Keywords:** aluminum alloys, extreme environments, in situ transmission electron microscopy, severe plastic deformation, space materials

## Abstract

Future human exploration of the solar system demands advanced materials capable of withstanding extreme environments, particularly exposure to solar energetic particle radiation. Current material selection criteria for space applications prioritize a high strength‐to‐weight ratio, high corrosion resistance and manufacturability, favoring age‐hardenable Al‐based alloys. However, conventional precipitation‐hardened Al alloys suffer from irradiation‐assisted dissolution of strengthening phases at doses as low as 0.2 displacements‐per‐atom (dpa), undermining their performance. Furthermore, these alloys develop radiation‐induced defects, such as dislocation loops and voids, even at low doses. This study presents a novel ultrafine‐grained (UFG) Al‐based alloy, designed using the crossover alloying concept and strengthened by T‐phase precipitates, featuring a chemically‐complex structure with 162 atoms in its unit cell composed of Mg_32_(Zn,Al)_49_. It is showed that T‐phase precipitates have exceptional radiation tolerance up to 24 dpa. Owing to the nanoscale UFG structure, dislocation loops are suppressed, and voids are only observed beyond 75 dpa. Microtensile tests up to 20 dpa confirm the preservation of mechanical performance under irradiation. The results underline the potential of this alloy as a radiation‐resistant, lightweight material for future space applications. Three key strategies enable this performance: (i) stabilization of a UFG microstructure, (ii) T‐phase precipitation featuring a highly negative Gibbs free energy and chemically‐complex giant unit cell, and (iii) precise process control to prevent grain growth during heat treatment and irradiation.

## Graphical abstract

A novel ultrafine‐grained aluminum crossover alloy exhibits unprecedented radiation resistance and mechanical stability under extreme irradiation doses up to 100 dpa. The exceptional resilience arises from thermodynamically stable T‐phase precipitates, enabling lightweight structural materials for next‐generation spacecraft and extraterrestrial applications where conventional Al alloys may fail under radiation exposure.

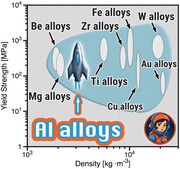

## 1 Introduction

Humanity's enduring drive to explore the unknown has propelled scientific progress and deepened our understanding of our place in the cosmos. Space exploration, a monumental challenge, relies on the multidisciplinary application of advanced technologies, rooted in one of humanity's oldest sciences: metallurgy. The role of this science in the human‐based exploration of space consists in the design and evaluation of the applicability of new materials for spacecrafts, satellites and space probes to survive within the harsh environment of space.^[^ ^1^, ^2^, ^3^, ^4^ ^]^ The knowledge accumulated over 70 years from multinational space programs has formed a list of materials requirements for application in extraterrestrial environments, considering the multiple degradation mechanisms that may operate synergistically while in‐service in space:^[^ ^2^, ^3^ ^]^ (i) high strength‐to‐weight ratio,^[^ ^5^, ^6^, ^7^, ^8^ ^]^ (ii) excellent thermal performance in a broader temperature range whilst in vacuum,^[^ ^7^, ^8^, ^9^, ^10^, ^11^, ^12^, ^13^ ^]^ (iii) high corrosion resistance to active monoatomic species (e.g. O) and to ionizing plasma,^[^ ^14^, ^15^, ^16^, ^17^ ^]^ (iv) easy manufacturability and repairability,^[^ ^18^, ^19^ ^]^ (v) costs,^[^ ^20^, ^21^ ^]^ and (vi) high radiation tolerance.^[^ ^1^, ^2^, ^3^, ^22^ ^]^ As a limiting factor, the first requirement calls for materials that are inherently lightweight as this is needed to minimise payload, fuel demands and low production costs. In terms of the interaction between both highly‐energetic particles and electromagnetic radiation with matter, our solar system can be considered an extreme environment for materials and the last requirement of high radiation tolerance is predominant considering long‐duration and long‐distance space missions with possible human settlement in extraterrestrial environments.^[^ ^2^ ^]^

In this context, the sources of radiation for both humans and materials within the solar system can be categorized as endogenous or exogenous. Endogenous radiation comprise the class that is generated within the interiors of a spacecraft, as for example, by a future small modular nuclear reactors (a.k.a. microreactors).^[^ ^23^, ^24^ ^]^ Exogenous source of radiation constitute of trapped radiation, cosmic rays, solar wind, solar flares and coronal mass ejections.^[^ ^4^, ^25^, ^26^ ^]^ These are critical for both humans and materials within the solar system. Space radiation is mostly generated by the Sun and its relationship with the solar cycles and its surrounding solar system is known as space weather.^[^ ^27^ ^]^ For low‐orbit missions, trapped radiation within the Earth's Van–Allen belts is problematic. These belts are magnetic fields that protects the Earth from the Sun's radiation via ion trapping, thus also containing a significant flux of highly energetic particles that can cause radiation damage in materials. For space‐missions beyond the Van–Allen belts and under space weather normal conditions, the total flux of solar energetic particles can reach 10^12^ ions cm^−2^  s^−1^ causing moderate damage to in‐service materials.^[^ ^25^, ^27^, ^28^ ^]^ Under abnormal conditions, solar flares and coronal mass ejections can significantly increase the radiation flux in a short‐period of time that may lead to severe radiation effects in spacecraft materials.^[^ ^3^ ^]^

Both endogenous and exogenous radiation sources within the context of space missions pose challenges for materials science with respect to the selection of structural materials for application in the extreme environment of the solar system.^[^ ^2^, ^3^ ^]^ Considering the strictly high strength‐to‐weight ratio as a major criteria for materials selection, Al is a preferential metal candidate and, in fact, Al‐based alloys are already used in several spacecraft and satellites structures with a dual purpose: to shield and to resist energetic particle and electromagnetic radiations.^[^ ^3^, ^4^ ^]^ Al‐based alloys are inherently lightweight due to their attainable low density and they can also be designed to achieve high levels of strength via precipitation hardening.^[^ ^29^, ^30^, ^31^ ^]^ The retention of such a high strength will be dependent upon the survivability of hardening precipitates under irradiation: if the radiation dissolves the precipitates, the alloy will lose the initially designed high strength.^[^ ^3^, ^32^ ^]^ “Ideal” materials for radiation environments are those that can preserve its initial properties upon impact of highly energetic particles with their crystalline lattices. Energetic particle irradiation can cause degradation via introduction of point defects into crystalline structures by displacing the lattice atoms from their equilibrium positions. In this context, displacements‐per‐atom (or dpa) is an average measure of how much lattice atoms are displaced from their lattice upon impinging atomic collisions.

Irradiation experiments in Al‐based alloys have so far shown both a tendency for saturation of displacement damage in a form of dislocation loops (causing irradiation‐induced embrittlement) and hardening phases dissolution (leading to alloy softening) at doses as low as 0.1 dpa considering commercial Al‐based alloys with micrometer‐sized grains.^[^ ^3^, ^32^ ^]^ Therefore, two major aspects are desirable for the application of novel Al‐based alloys as future space materials, which requires alloy design: a hardening phase capable of resisting high doses of irradiation and an alloy matrix capable of resisting the development of displacement damage in the form of dislocation loops and voids. We demonstrate in this work the solution to the problem via the synthesis of a stable ultra‐fine grained (UFG) microstructure of a novel aluminum crossover alloy. Recently invented via the combination between two distinct classes of aluminum alloys, the AlMg and AlZnMg(Cu) alloys (AA5xxx/AA7xxx), the aluminum crossover alloys were found to be hardenable via precipitation of a highly‐concentrated and chemically‐complex ternary intermetallic superstructure: the T‐phase with chemical formula Mg_32_(Zn,Al)_49_.^[^ ^33^ ^]^ For simplicity, the herein investigated crossover alloy – Al‐5.34Mg‐1.56Zn‐0.26Cu‐0.04Ag in at.% – is referred to AlMgZnCuAg throughout the article. Usually, nanocrystalline or UFG Al‐based alloys tend to fast recrystallise at low temperatures.^[^ ^4^, ^34^ ^]^ We prove that suitable heat treatment procedures enable the UFG structure of our Al‐based crossover alloy to both precipitate the T‐phase and preserve its average matrix grain size within the nanoscale upon large doses of energetic particle irradiation. Heavy ion irradiations with in situ Transmission Electron Microscopy (TEM) were performed at the MIAMI facility using a 300 keV Ar^+^ ion beam line.^[^ ^35^ ^]^ This methodology allowed a real‐time assessment and direct microstructural monitoring of the radiation effects. Using the same irradiation facility, but with a 600 keV Ne^+2^ ion beam, we subjected specimens to irradiation up to a peak dose of 20 dpa where the mechanical properties were then evaluated using microtensile testing.

## 2 Results and Discussion

**Figure 1:**
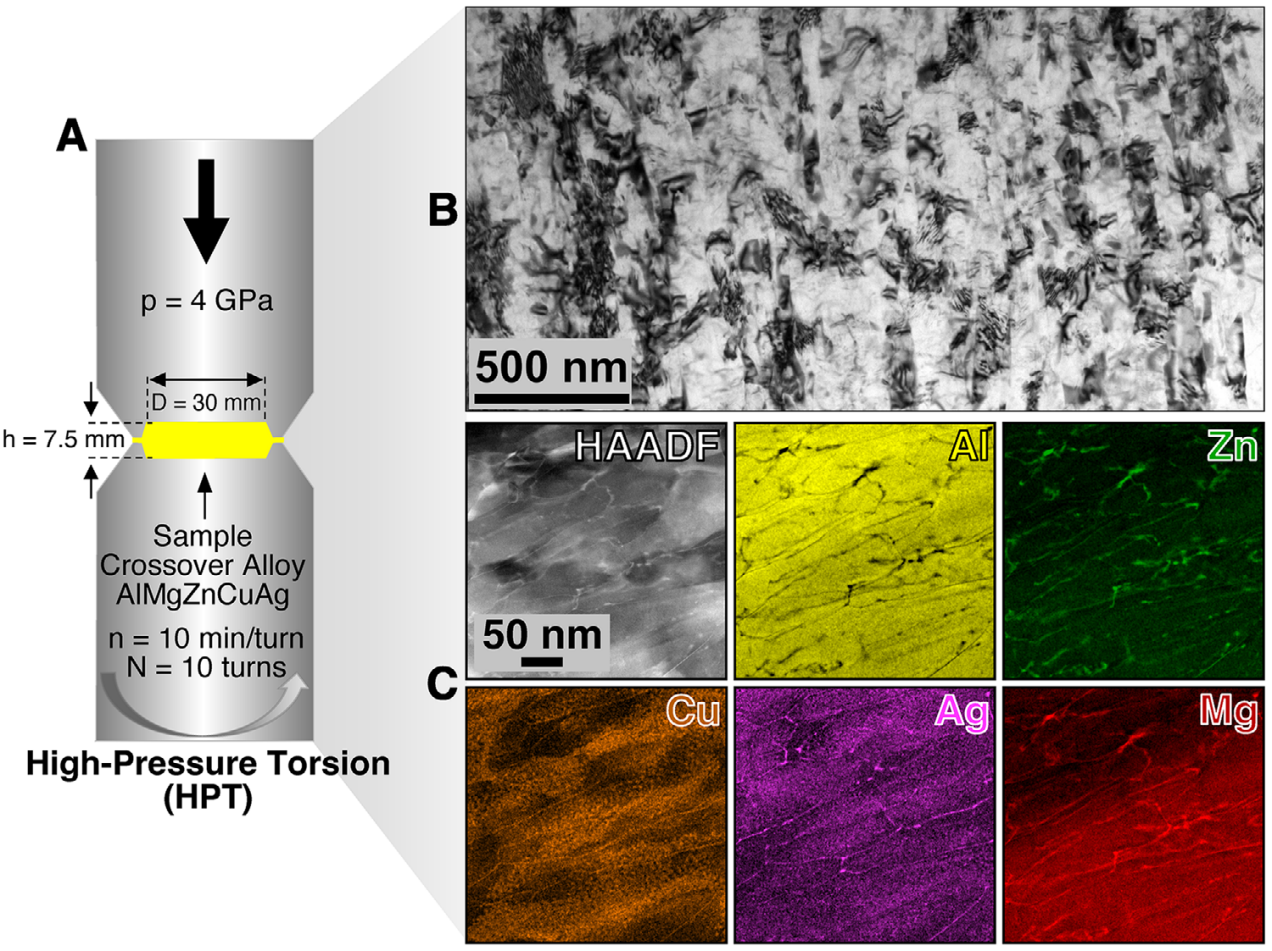
Synthesis of the UFG aluminum crossover alloy. A) To achieve an UFG microstructure from the bulk AlMgZnCuAg crossover alloy, the technique of HPT was used. B) After processing, BFTEM revealed a UFG microstructure. Local nanochemistry analysis revealed segregation of all alloying elements to the grain boundaries, but no T‐phase precipitates in the as‐processed condition as shown in the STEM‐EDX mapping in (C).

The procedure for obtaining the UFG AlMgZnCuAg crossover alloy is shown in **Figure** **1**A. After casting and High‐Pressure Torsion (HPT), a characteristic UFG microstructure was observed as shown in the Bright‐Field TEM micrograph (BFTEM) in Figure 1B. Scanning Transmission Electron Microscopy (STEM) with coupled Energy‐Dispersive X‐ray spectroscopy (EDX) assessment of the alloy in the as‐processed condition after HPT is also presented in Figure 1C. No T‐phase precipitation was observed, although the goal to achieve grains confined within the nanometer‐scale was successful. The average grain length was estimated to be 294.3 ± 2.6 nm whereas the average grain width was estimated to be 91.4 ± 36.6 nm with the errors representing the standard deviations.^[^ ^36^ ^]^ Mg, Zn, Cu, and Ag segregation along the grain boundaries was noted after alloy synthesis. The absence of T‐phase precipitates suggested the need for heat‐treatment to enable its formation. The challenge here is to overcome the well‐known low‐temperature recrystallization of UFG Al‐based alloys.^[^ ^34^ ^]^

**Figure 2:**
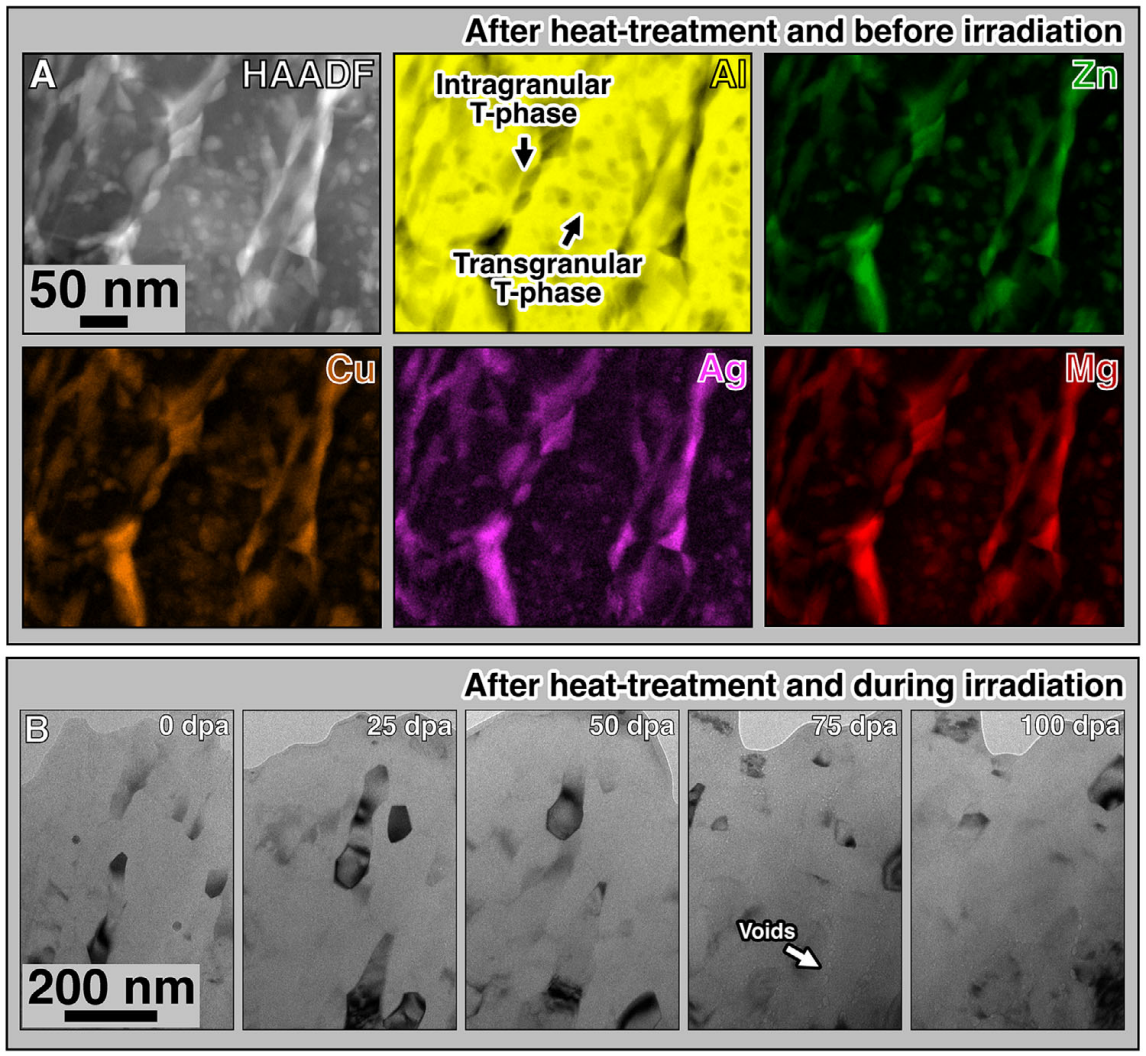
Alloy's stability under irradiation. Nucleation of T‐phase precipitates in the UFG AlMgZnCuAg crossover alloy was observed after heat‐treatment using a ramp of 10 K min^−1^ up to 506 K (A). The microstructural evolution of the UFG AlMgZnCuAg crossover alloy as monitored in situ within the TEM is shown in the set of underfocused BFTEM micrographs in (B) from 0 to 100 dpa. The alloy's microstructure neither exhibit formation of dislocation loops nor grain growth at a maximum dose of 100 dpa. Voids are only observed to form at around 75 dpa.

For the UFG AlMgZnCuAg crossover alloy investigated in this work, we found that a controlled heat‐treatment using a ramp rate of 10 K min^−1^ up to 506 K was sufficient to promote nucleation and growth of T‐phase precipitates whilst recrystallization was not observed. The set of STEM‐EDX elemental maps in **Figure** **2**A shows the microstructure of our alloy after the chosen heat‐treatment route. It is worth emphasising that, the Supporting Information 1 shows the resulting microstructures after heat‐treatment in different ramp rates. T‐phase precipitates are observed not only at transgranular positions, but also along intragranular positions. The average size of T‐phase precipitates after this heat‐treatment was measured to be 6.7 ± 0.7 nm.^[^ ^36^ ^]^ STEM‐EDX measurements revealed no chemical difference between precipitates at transgranular or intragranular sites. In addition, according to our previous work, only T‐phase precipitates were identified in this UFG AlMgZnCuAg crossover alloy as measured via both grazing‐incidence X‐ray diffraction (GI‐XRD) and differential scanning calorimetry (DSC).^[^ ^36^ ^]^ These results also validate another study on a bulk aluminum crossover alloy with similar chemical composition.^[^ ^37^ ^]^ By pinning the grain boundaries at the nanoscale and stabilising the initial UFG microstructure, the T‐phase was observed to prevent the phenomenon of recrystallization at temperatures up to 553 K.^[^ ^36^ ^]^ Further detailed characterization of grain‐boundaries and T‐phase precipitates can be found in the Supporting Information file. Therefore, the T‐phase precipitation potential of the UFG AlMgZnCuAg crossover alloy was herein demonstrated. It is worth noting that various heat‐treatment conditions have been tested within this project and it was observed that recrystallization happened only at much faster heating rates for the UFG AlMgZnCuAg crossover alloy as shown in the Supporting Information file.

Irradiation experiments were performed up to extreme doses of 100 dpa, which represents the average value over the whole specimen thickness. The BFTEM micrographs in Figure 2B were acquired during the in situ TEM irradiations of the alloy after heat treatment. Two important conclusions can be drawn from the results of the irradiation tests. First, neither formation nor accumulation of irradiation‐induced dislocation loops is noted even though the alloy was real‐time monitored using a multi‐beam condition in BFTEM. This is opposed to our previous observations in a coarse grained Al‐based crossover alloy where numerous dislocation loops formed and accumulated and resulted in irradiation‐induced embrittlement typically observed in metals and alloys.^[^ ^3^ ^]^ Second, no irradiation‐induced grain‐growth was noted. As recently reviewed in literature,^[^ ^38^ ^]^ this result is of a particular interest as many metallic nanocrystalline alloys subjected to ion irradiation severely suffer from this effect already at lower doses than herein tested. Voids were detectable within the alloy's microstructure at doses higher than 75 dpa as shown in Figure 2B. This is a remarkable achievement for this novel UFG AlMgZnCuAg crossover alloy when considering the envelope of operation of space materials in long‐duration space missions:^[^ ^4^ ^]^ 75 dpa is a far extrapolated dose for space materials. At the same time, it is also a remarkable resistance to displacement damage type radiation effects considering next generation of fission and fusion nuclear reactors,^[^ ^38^, ^39^ ^]^ which presents more aggressive irradiation conditions than the space.

Both the absence of dislocation loops and grain growth already suggest that the novel UFG AlMgZnCuAg crossover alloy exhibits an outstanding level of radiation tolerance to displacement damage. It is important emphasising that some nanocrystalline and UFG alloys – as for example some select metals like W and Fe^[^ ^40^, ^41^ ^]^ as well as more recently with complex high‐entropy alloys^[^ ^42^, ^43^, ^44^, ^45^, ^46^ ^]^ – are known to exhibit higher radiation tolerance against displacement damage formation and accumulation within their microstructure due to high sink‐strength permitted by polycrystalline grains within the nanometre range,^[^ ^38^, ^47^, ^48^ ^]^ but up‐to‐date and to the best of our knowledge, this has not yet been demonstrated in the metallurgy of lightweight aluminum alloys. Nevertheless, to completely assess the radiation tolerance of the novel UFG AlMgZnCuAg crossover alloy, final proof for the survivability of the T‐phase precipitates under irradiation needs to be fully disclosed.

It is worth emphasising that the dissolution of hardening precipitates under irradiation occurs via ballistic mixing (BM).^[^ ^3^, ^32^ ^]^ The ballistic impact of a highly energetic particle within the crystalline lattice of an alloy generates a cascade of point defects, raising local temperature for an ultra‐short period of time which results in a complete local chemical reorganization of atoms in a crystal, yielding the dissolution of hardening phase particles. Only a limited number of studies exist on BM‐assisted dissolution of age‐hardening precipitates in Al‐based alloys. The commercial Al‐based alloy grade AA6061‐T6 was shown to be susceptible to BM‐assisted dissolution of its hardening phase – the Mg_2_Si known as β‐phase – which did not survive doses up to 0.2 dpa when exposed to proton beams with energies between 600–800 MeV.^[^ ^32^ ^]^ Lohmann et al. results were both timely and independently validated by Singh et al.^[^ ^49^ ^]^ It is worth noting that the AA6061‐T6 is commercially significant and widely used as structural material for aerospace applications. Severe radiation effects have been also reported to other commercial Al‐based alloys,^[^ ^50^, ^51^, ^52^, ^53^, ^54^, ^55^, ^56^ ^]^ which motivated scientific research to develop novel Al‐based age‐hardenable alloys capable of resisting the deleterious effect of radiation exposure. Opposed to Lohmann et al. and Singh et al.,^[^ ^32^, ^49^ ^]^ Flament et al. irradiated the AA6061‐T6 with heavy‐ions with high energies (4 MeV Au and 2 MeV W ions).^[^ ^57^ ^]^ These authors report partial dissolution of Mg_2_Si precipitates at around 95 dpa and complete dissolution after 165 dpa, but have not investigated the effects of irradiation on the hardening phases at lower doses (⩽1 dpa). Therefore, whether the Mg_2_Si dissolve at lower doses and re‐precipitates at higher doses further research is pending clarification.

**Figure 3:**
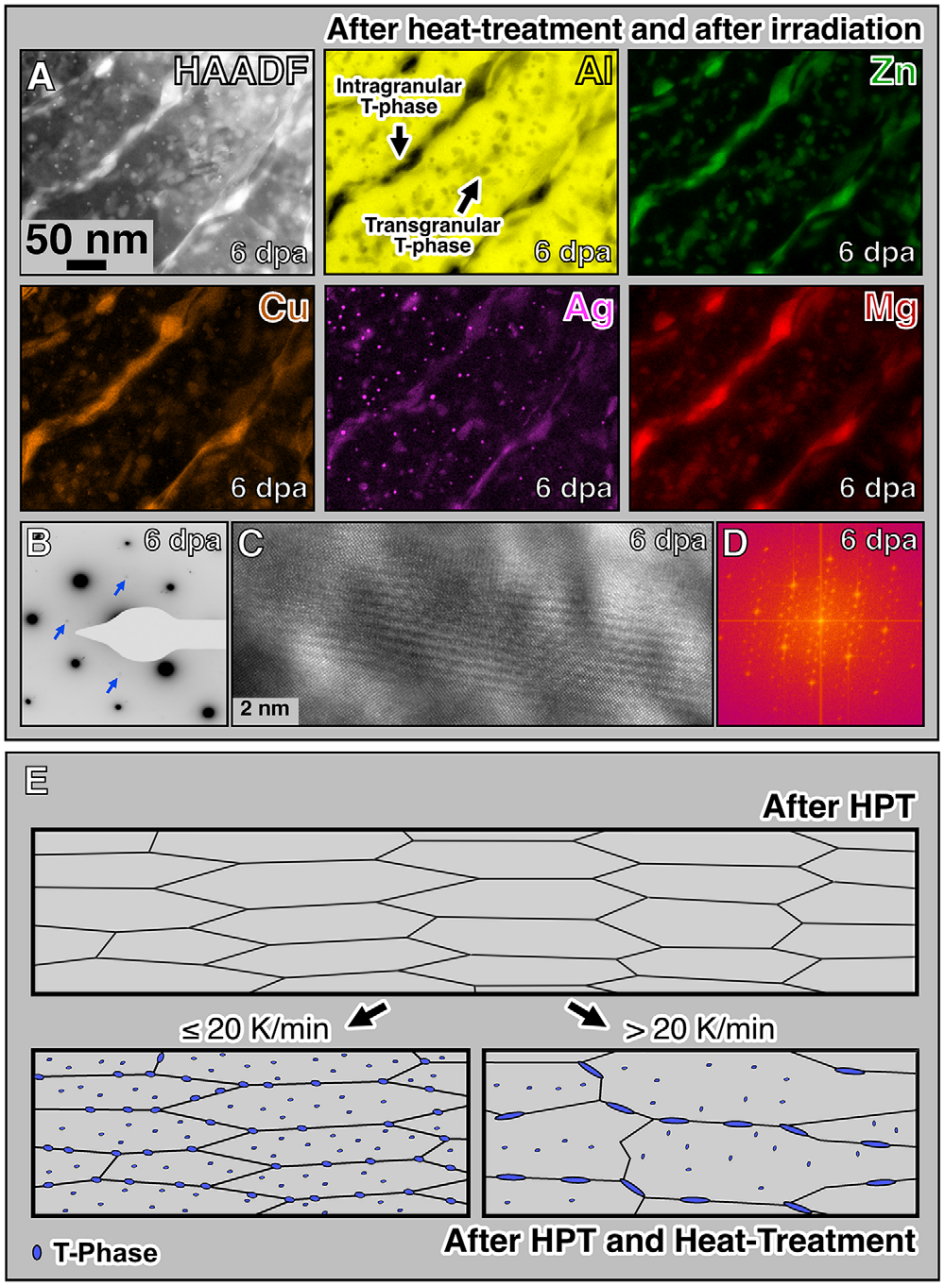
Alloy's stability under irradiation (post‐irradiation examination). Post‐irradiation methodologies using conventional and analytical electron‐microscopy were used to investigate the origins of the alloy's stability under irradiation. A) STEM‐EDX mapping and B–D) SAED, HRTEM and FFT, respectively, show that T‐phase precipitates are stable and did not dissolve at the exemplary dose of 6 dpa, which is 6^[^ ^3^ ^]^ and 30^[^ ^32^ ^]^ times higher dose than previous reports on irradiation‐assisted dissolution of hardening phases in bulk Al‐based alloys. The schematics in (E) exhibit microstructural differences in the UFG alloy before and after a heat treatment, establishing a new alloy design strategy to achieve high radiation tolerance. T‐phase precipitates are prone to nucleate, grow and stabilise the structure.

Preliminary research demonstrated that the T‐phase has a superior Radiation Survivability Level (RSL) of 1 dpa when compared with other hardening phases in conventional Al‐based alloys.^[^ ^3^ ^]^ T‐phase precipitates in a Al‐5.30Mg‐1.42Zn (at.%) micrometre‐grain sized crossover alloy survived up to 1 dpa using heavy ions with 100 keV Pb^+^. A possible higher RSL for T‐phase precipitates in the UFG Al‐based crossover alloy is a hypothesis to be tested in this work. As these precipitates can be hardly seen using the multi‐beam and lower‐magnification BFTEM condition due to their small sizes, STEM‐EDX mapping as shown in **Figure** **3**A were acquired after irradiation around 6 dpa. These images demonstrate that T‐phase survived at this irradiation dose. This has been additionally proven by subsequent post‐irradiation assessment using Selected‐Area Electron Diffraction (SAED) pattern, High‐Resolution TEM (HRTEM) with derived FFT, respectively in Figure 3B–D. For reference, the observed superlattice reflections (more visible in the FFT) agree well with previous identification of T‐phase precipitates.^[^ ^33^, ^37^, ^58^, ^59^ ^]^ Given the observations made so far, the results shown in this research serve as a guidance to elaborate a new alloy design strategy for novel UFG Al‐based alloys to be used in extreme environments. This is described in the schematics presented in Figure 3E: After processing via HPT the alloys' potential is not yet fully exploited. A suitable heat treatment must be applied in order to precipitate T‐phase both along the grain boundaries and at transgranular positions. Using this alloy design strategy, the mechanisms of high radiation tolerance can be harnessed. The presence of homogeneously distributed nanoprecipitates in higher volumetric fraction compared with commercial Al‐based alloys^[^ ^3^ ^]^ as well as the fact that the alloy confines its grain size within the nanoscale suggest a significant ability for absorption of radiation‐induced point defects without material degradation, thus preventing the manifestation of extended radiation effects (except for voids at very high doses of 75 dpa).

**Figure 4:**
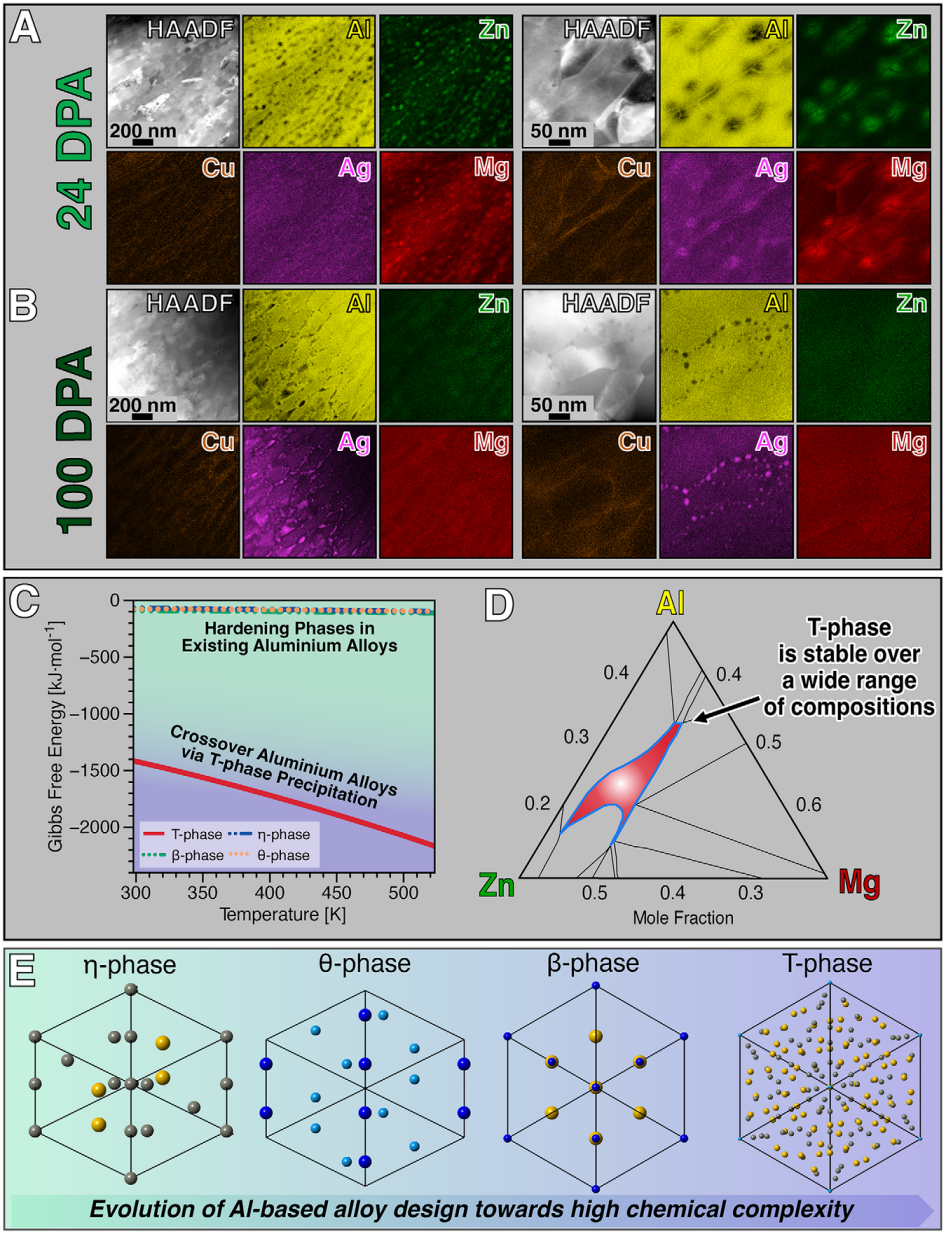
Radiation Survivability Level of T‐phase precipitates and thermodynamic origins of their high radiation tolerance. A survey using STEM‐EDX mapping in the post‐irradiated specimens shows that T‐phase precipitates surviving up to a dose of 24 dpa, as denoted in (A). After 24 dpa, the precipitates began to (progressively) dissolve and at the dose of 100 dpa, (B), no T‐phase precipitates were detected in the UFG AlMgZnCuAg crossover alloy. Radiation‐induced precipitation of pure Ag nanoprecipitates is noted only at doses around 100 dpa. Thermodynamic calculations in plot (C) show that the Gibbs free energy for T‐phase precipitates is significantly lower compared with hardening precipitates within the existing Al‐based alloys, pointing to superior thermodynamic stability. The T‐phase is found to be stable over a wide range of chemical ratios as shown in the ternary equilibrium phase diagram in (D) calculated at both 298 K and 1 bar. E) The crystal structures of the (Al_2_Cu) θ‐phase, (Mg_2_Zn) η‐phase, (Mg_2_Si) β‐phase and (Mg_32_(Zn,Al)_49_) demonstrate that chemical complexity is a distinct characteristic of the T‐phase precipitates tailoring the radiation resistance of the UFG AlMgZnCuAg crossover alloy.

Post‐irradiation investigations beyond 6 dpa were performed to assess the RSL of T‐phase precipitates within the UFG AlMgZnCuAg crossover alloy. **Figure** **4**A,B shows the STEM‐EDX maps taken at low and high magnification after 24 and 100 dpa, respectively. We discovered that the RSL threshold of T‐phase precipitates within the UFG AlMgZnCuAg crossover alloy is 24 dpa. T‐phase was observed to be fully dissolved at 100 dpa. To the best of our knowledge, a RSL of 24 dpa is a new record scored among all known hardening precipitates tested under irradiation so far. This is 24 times higher compared to our previous record of 1 dpa in the coarse grained Al‐based crossover alloy^[^ ^3^ ^]^ and 120 times higher than Mg_2_Si in conventional AA6061‐T6 as tested by Lohmann et al.^[^ ^32^ ^]^

Thermodynamic calculations were used to shed light on the stability of the major precipitate phases in commercial aluminum alloys in comparison with the herein introduced novel UFG AlMgZnCuAg crossover alloy. Figure 4C reveals interesting and important feature of Al‐based crossover alloys: the T‐phase's Gibbs free energy exhibit the highest negative value known so far among the hardening precipitates within the whole spectrum of commercial aluminum alloys, i.e. (Mg_2_Zn) η‐, (Al_2_Cu) θ‐, (Mg_2_Si) β‐phase. A large negative Gibbs potential is characteristic of high phase stability in metallurgy.^[^ ^60^ ^]^ The crystal structures of all these hardening phases as well as the T‐phase are shown in Figure 4E. T‐phase exhibits both distinct largely negative Gibbs potential and chemically‐complex crystal structure when compared to other precipitates in Al‐based alloys. Its largely negative Gibbs free energy stems from both high entropy and enthalpy as shown in the thermodynamic calculations presented in the Supporting Information file 5. In fact, Bergman and Pauling et al. discovered in the late 1950s,^[^ ^61^ ^]^ that T‐phase has a characteristic unit cell structure comprising 162 atoms per cube. BM‐assisted dissolution of precipitates requires that the incoming and highly‐energetic atoms promote dissociation of constituents via atomic displacements in an uncontrolled manner that inevitably leads to destruction of the crystalline state. Our results obtained herein with the T‐phase suggests that − below its RSL − both atomic displacements and stoichiometry changes can take place without major chemical changes in its bulk crystal structure as promoted by atomic displacements during the events of irradiation (Figure 4D). Song et al.^[^ ^62^ ^]^ also reported on the specific thermodynamics of multiple T‐phase variants when comparing to the η‐phase (in AA7xxx series alloys), which also correlate to the observed high‐radiation tolerance herein reported. Evidence herein presented may indicate that the origins of high radiation tolerance of T‐phase precipitates resort – and can be tailored – to its unique thermodynamic state which translates to high phase‐stability in extreme environments promoted by chemical complexity and high chemical disorder tolerance, the latter in‐line with the findings presented by Shimizu et al.^[^ ^63^ ^]^

It has been previously demonstrated that the T‐phase can dissolve both Cu and Ag in its crystal structure contributing both its thermodynamic stability and to an enhancement of mechanical properties.^[^ ^37^, ^59^ ^]^ Upon the irradiation experiments, Ag nanoprecipitates do form at 6 dpa (see Figure 3A), dissolve at 24 dpa (see Figure 4A), and re‐precipitate back preferentially into transgranular positions at a dose around of 100 dpa (see Figure 4B). It is worth noting that Mg, Zn and Cu – major constituents of the T‐phase – are not affected in a similar manner. It is worth to mention that the vast majority of Ag is found within the T‐phase before and during irradiation up to 24 dpa, thus the nucleation of Ag nanoprecipitates did not harm the stability of the T‐phase under irradiation. Additions of Ag into the crossover alloy were found to be of high importance for the thermodynamic stability of the T‐phase.^[^ ^59^ ^]^ Therefore, the addition of Ag is required to support nucleation of the T‐phase and its irradiation resistance.

So far, the irradiation experiments indicate that neither grain growth nor the development of microstructural defects like dislocation loops or stacking fault tetrahedral were observed up to 100 dpa. STEM‐EDX assessment also revealed that the T‐phase is stable up to 24 dpa. Moreover, only at high doses such as 75 dpa, were voids detected in the matrix. To shed light on the impact of ion irradiation in the mechanical properties of this novel UFG AlMgZnCuAg crossover alloy, microtensile experiments were also performed. Here, we exposed samples to a 600 keV Ne^2 +^ ion beam (i.e. ex situ TEM) in the MIAMI‐2 system. By sequentially irradiating both sides of the microtensile specimens with the 600 keV Ne^+2^ ion beam, a nearly uniform (“top‐hat”) damage profile was achieved across the entire specimen thickness, ensuring that the mechanical response reflects the fully irradiated volume.

**Figure 5:**
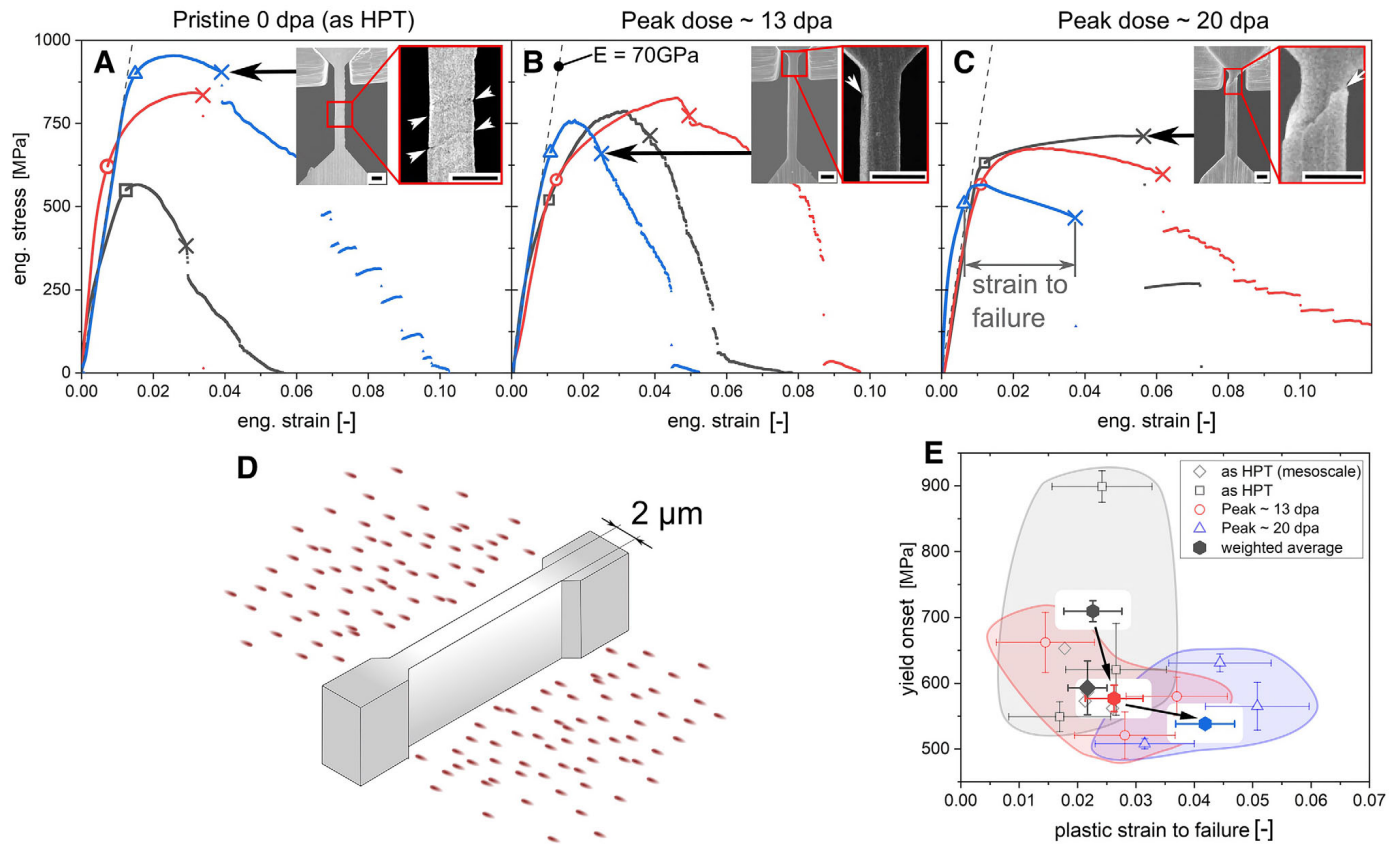
Mechanical response before and after irradiation. Engineering stress‐strain curves of (A) as‐HPT as well as after irradiation to peak (B) 13 dpa and (C) 20 dpa, respectively. Insets show the in situ SEM micrographs of specimens right at failure with white arrows depicting the position of the determined failure feature. All micron bars correspond to 2 µm. D) Schematic of the Ne irradiation process of the pre‐thinned lamella. E) Data summarization of yield onset and strain to failure for all microtensile samples (open symbols) as well as weighted average (filled symbols) for the respective states. For comparison, the yield onset and strain to failure of mesoscale as‐HPT specimens was added as well (rotated grey‐square).

**Figures** **5**A,B,C show the results of the micromechanical testing for the pristine (0 dpa), 13 dpa, and 20 dpa samples, respectively. The irradiation methodology that allowed the micromechanical ttesting of the alloy after irradiation is shown in Figure 5D. The microscale tensile experiments of the as‐HPT unirradiated state show (on average) a higher yield onset of 709 ± 16 MPa than the mesoscale specimen at 593 ± 41 MPa. However, the plastic strain‐to‐failure is nearly identical at 2.3 ± 0.5% for the microscale and 2.2 ± 0.3% for the mesoscale. Considering the single highest value of the microscale testing as an outlier, the average yield onset for the as‐HPT and unirradiated sample was 556 ± 21 MPa at a similar strain‐to‐failure of 2.3 ± 0.6%. After a peak dose of 13 dpa, the average yield onset was estimated to be 577 ± 20 MPa at a strain‐to‐failure of 2.2 ± 0.3%. Therefore, the results from the mesoscale and microscale specimens of the as‐HPT and unirradiated state as well as the microscale specimens of the 13 dpa (peak) irradiated state can all be considered to fail in a similar metallurgical regime. It is worth emphasising that the values of the microscale specimens should be evaluated considering the known potential for grain boundary embrittlement due to Ga implantation.^[^ ^64^, ^65^, ^66^ ^]^ The overall agreement with the mesoscale specimens indicates that any potential impact of the FIB fabrication process on the mechanical properties (if any) is negligible and not detectable within the existing experimental accuracy (Table 1). Figure 5E shows a compilation of the results obtained with both mesocale and microtensile testing in both pristine and irradiated samples. These results are also consistent (Figure 5) with previous mechanical data on UFG 7075 (Al–Zn–Mg) alloy reported by Ma et al.^[^ ^67^ ^]^

**Table 1:** Assessment of the mechanical properties before and after irradiation.

	Yield onset [MPa]	Strain‐to‐failure [%]	Testing method
Pristine (as‐HPT)	709 ± 16	2.3 ± 0.5%	Mesoscale testing
Pristine (as‐HPT)	556 ± 21	2.3 ± 0.6%	Microtensile testing
13 dpa (peak)	577 ± 20	2.2 ± 0.3%	Microtensile testing
20 dpa (peak)	538 ± 7	4.2 ± 0.3%	Microtensile testing

On the other hand, the higher irradiation dose – which peaks at around 20 dpa – led to a slight reduction of yield onset to 538 ± 7 MPa, but a significant increase in strain‐to‐failure (4.2 ± 0.3%). Based on the data from microtensile experiments, it seems that the irradiation damage up to 13 dpa does not significantly alter the mechanical response of the pristine UFG AlMgZnCuAg crossover alloy. At peak 20 dpa – closer to the RSL of T‐phase precipitates – a slight reduction in yield onset indicates that the T‐phase precipitates start to dissolve. However, this is *commensurate* with a significant increase in strain‐to‐failure, suggesting that the overall tolerance to mechanical failure remains high, even after such very high irradiation dose levels.

## 3 Conclusion

The design of new materials for stellar‐radiation environments currently present several challenges mainly with respect to radiation resistance. The criteria of high strength‐to‐weight ratio is mandatory for space programs, limiting the choices to metals exhibiting lower densities. In this research, we introduced a new alloy design methodology that led to the synthesis of a new UFG AlMgZnCuAg crossover alloy with high radiation tolerance. This alloy features unique T‐phase precipitates with an estimated RSL of 24 dpa, a new irradiation dose record for aluminum alloys in extreme environments. In addition, due to the UFG microstructure, the alloy has not exhibited any detectable dislocation loops as a result of irradiation, and voids were only observed at a dose of 75 dpa. We have shown that by tailoring the thermodynamics at the atomic level, new materials can be designed to sustain radiation levels that exceed even the exogenous conditions found in the solar system. Microtensile experiments reveal no negative impact of irradiation in the mechanical response of this new alloy up to 20 dpa: neither irradiation‐induced hardening nor embrittlement were detected. Further research is required to unveil the full mechanisms by which T‐phase precipitates survive irradiation in such aggressive conditions, specially considering its unique thermodynamics when compared with conventional hardening phases in existing commercial alloys.

## 4 Experimental Section

### Synthesis of the Alloy and Post‐Synthesis Processing

A novel UFG Al‐based crossover alloy within the quinary system of Al–Mg–Zn–Cu–Ag was synthesized using a vacuum induction melting furnace. Casting was performed in a Cu mould and the elemental composition was measured to be Al‐5.34Mg‐1.56Zn‐0.26Cu‐0.04Ag in at.% using Optical Emission Spectroscopy. After casting, the alloy slabs were homogenized at both 733 and 743 K followed by a machining step to obtain a disk with 6.5 mm thickness and 30 mm in diameter. The last step was necessary to fit the anvil for the HPT application. To obtain a UFG structure, HPT was used with 4 GPa of pressure, 10 turns comprising 10 min revolution^−1^ for each turn. To investigate the microstructural stability and precipitation behavior of the UFG Al‐based crossover alloy upon heating and irradiation, samples for Scanning and Transmission Electron Microscopy (STEM/TEM) were prepared from the outer radius of HPT disk to ensure a microstructure with uniform distribution of grain sizes. Different heat treatment strategies were applied and studied: as‐processed via HPT and at 5, 10, and 20 K min^−1^ up to 506 K.

### Sample Preparation for Electron Microscopy

Thin‐foil for electron microscopy were prepared from the as‐processed condition. Samples were ground to a thickness between 80 and 100 µm. Disks with 3 mm (diameter) were punched from the foil and subjected to twin Jet Electropolishing using a solution of 25% nitric acid and 75% methanol (in volume) at a temperature range from 243 to 248 K with an electric potential of 12 V until perforation. After electropolishing, specimens were washed in three sequential pure methanol baths and left to dry in the air.

### In Situ TEM Annealing

In situ TEM annealing was carried out to investigate the microstructural response and stability of the UFG Al‐based alloy in addition to evaluate its precipitation behavior. For these experiments, a Protochips FUSION MEMS chip‐based holder in a Thermo Fisher Talos F200X S/TEM was used followed by a sample preparation procedure described in literature.^[^ ^68^ ^]^ In addition to the MEMS experiments and prior to irradiation, the as‐processed samples were subjected to heat‐treatment using a Gatan double‐tilt heating holder model 652. The heat‐treatment was performed at a ramp rate of 10 K min^−1^ up 506 K in order to allow the precipitation of the T‐phase (see the Supporting Information 2).

### In Situ TEM Ion Irradiation

In situ TEM heavy ion irradiations were carried out in the MIAMI‐2 facility located at the University of Huddersfield.^[^ ^35^ ^]^ The irradiation experiments were performed using a 300 keV Ar^+^ ion beam. During the irradiation experiments, samples were monitored using a Gatan Oneview 4k camera coupled with a Hitachi H9500 TEM operating at 300 keV. The flux was measured at the specimen position using a current metering rod and it was estimated to be 7.74 × 10^13^ ions cm^−2^ s^−1^ with an empirical error of 10%. The Stopping and Range of Ions in Matter (SRIM) code^[^ ^69^ ^]^ was used to convert fluence to an equivalent dose in displacement‐per‐atom (dpa) following a procedure suggested by Stoller et al.^[^ ^70^ ^]^ Under the ion irradiation conditions presented in this work, the maximum fluence achieved during the experiments was 2.30 × 10^17^ ions cm^−2^, which corresponds to an equivalent dose average of 100 dpa. To be consistent with previous works,^[^ ^3^ ^]^ 40 eV was used as displacement energy for aluminum, which is higher than the 25 eV present in SRIM as default. Nevertheless, if 25 eV is used for the fluence‐to‐dpa conversion using the Stoller et al.^[^ ^70^ ^]^ procedure, both calculated dose levels are 100 dpa on average (see Supporting Information 3). This ion irradiation set‐up was found to be a convenient way to simulate the displacement cascades generated by the primary knock‐on atoms (PKA) in Al when subjected to collisions with highly‐energetic proton beams emitted by the Sun,^[^ ^3^, ^71^ ^]^ and without radioactive activation of the UFG Al‐based crossover alloy.

### Pre‐ and Post‐Irradiation Characterization Methodology

Pre‐ and post‐irradiation characterization was carried out using both a Thermo Fisher Scientific Talos F200X and a Thermo Fisher Titan 30–800 scanning transmission electron microscopes. For investigations high annular dark field (HAADF), bright‐field (BF‐TEM), high‐resolution (HRTEM) and energy‐dispersive X‐ray spectroscopy (EDX) measurements were carried out.

### Ex Situ TEM Ion Irradiation for Micromechanical Testing

To enable microtensile testing after irradiation, two specimens were irradiated at the MIAMI‐2 facility using an ex situ TEM configuration (i.e., without electron beam monitoring). A 600 keV Ne^2 +^ ion beam with a flux of approximately 4.40 × 10^12^ ions cm^−2^  s^−1^ was employed. To achieve a uniform damage profile across the 2 µm thickness of the specimens, both sides were irradiated to fluences of (i) 5.15 × 10^16^ ions cm^−2^ and (ii) 8.24 × 10^16^ ions cm^−2^. As shown in the Supporting Information file 4, fluence‐to‐dpa conversion of these fluencies indicate these dose levels correspond to peak doses of 13 and 20 dpa, respectively.

### Micromechanical Testing

The micromechanical specimens were fabricated on 2 × 3.5 mm^2^ pieces which were polished into a 5° inclined wedge‐shaped sample. For irradiation, one section was pre‐thinned to a thickness of 2 µm using focused Ga+ ion beam milling (FIB, LEO 1540XB, Carl Zeiss AG, Oberkochen, Germany) with 30 kV acceleration voltage and 2 nA current. Using double‐sided 600 keV Ne ion irradiation within the MIAMI‐2 facility, the complete volume of the micromechanical sample is expected to be exposed. Dogbone shaped tensile specimens were fabricated with an approximate cross‐section of 1.5 × 1.5 µm^2^ and a gauge length of 15 µm. The specimens were fabricated with the same FIB station, using final quick polishing passes at 500 pA as a tradeoff between smooth surface finish and unwanted Ga implantation possibilities.

The micromechanical tensile experiments were performed in situ inside a scanning electron microscope (LEO1530, Carl Zeiss AG, Oberkochen, Germany) equipped with a FemtoTools nanomechanical testing device (FT‐NMT04, FemtoTools AG, Buchs, Switzerland). The tests were carried out in a displacement‐controlled mode at 15 nm s^−1^ (engineering strain rate around 10^−3^) without any inherent frame stiffness correction. To collect raw data from the experiments, a MEMS load sensor with a maximum load of 20 mN and a custom tungsten tip – which was FIB‐fabricated to a 45° side‐walled gripper shape – was used. The engineering strain was evaluated from the in situ images based on a neural network assisted feature segmentation algorithm^[^ ^72^ ^]^ and the engineering stress was evaluated classically by division through the initial cross‐section of each specimen, respectively. Yield onsets were determined by individual 0.2% shifted linear slopes fitted within the range of 150–400 MPa in analogy to macroscopic measurements; all error estimates are based on uncorrelated input quantities,^[^ ^73^ ^]^ incorporating strain errors based on 3 px image deviations, load noise levels of 0.1 µN and cross‐sectional area deviations based on the standard deviation of 10 individual measurements.

### Thermodynamic Calculations

The thermodynamic data presented in the curves in Figure 4C,D were calculated using the thermochemical software FactSage 8.0 and the FTlite database.^[^ ^74^ ^]^ As the intermetallic T‐phase has no strict stoichiometric value for Zn or Al, we determined the composition of the T‐phase in our alloy based on the STEM‐EDX elemental quantification measurements reported in Figure 2A after artificial aging temperature at 506 K. Then, this obtained composition was used to calculate the thermodynamic potentials as a function of temperature within the range from 273 K to 523 K. Furthermore, the variation of Gibbs free energy as a function of temperature of essential hardening phases in different Al‐based alloys were drawn comparatively. Therefore, we selected θ‐phase (Al_2_Cu), β‐phase (Mg_2_Si) and η‐phase (MgZn_2_) for 2xxx, 6xxx, and 7xxx series alloys, respectively. The Supporting Information file 5 contains both enthalpy and entropy calculations for given hardening phases.

## Conflict of Interest

The authors declare no conflict of interest.

## Supporting information



Supporting Information

## Data Availability

The data that support the findings of this study are available from the corresponding author upon reasonable request.
