# Supplementary material for: Radiation‐Resistant Aluminum Alloy for Space Missions in the Extreme Environment of the Solar System
Source: Adv Mater. 2025 Dec 15;38(20):e13450. doi: 10.1002/adma.202513450 (PMC13054116; doi:10.1002/adma.202513450)
Supplement: Supplementary file 1 — Supporting Information [file ADMA-38-e13450-s001.pdf]

Radiation-resistant aluminium alloy for space missions in the extreme environment of the solar system

P.D. Willenshofer, M.A. Tunes *et al.*

Supplemental information for the main paper

(2025)

Supplemental Information 1 – Microstructure after annealing up to 506 K (STEM-EDX)

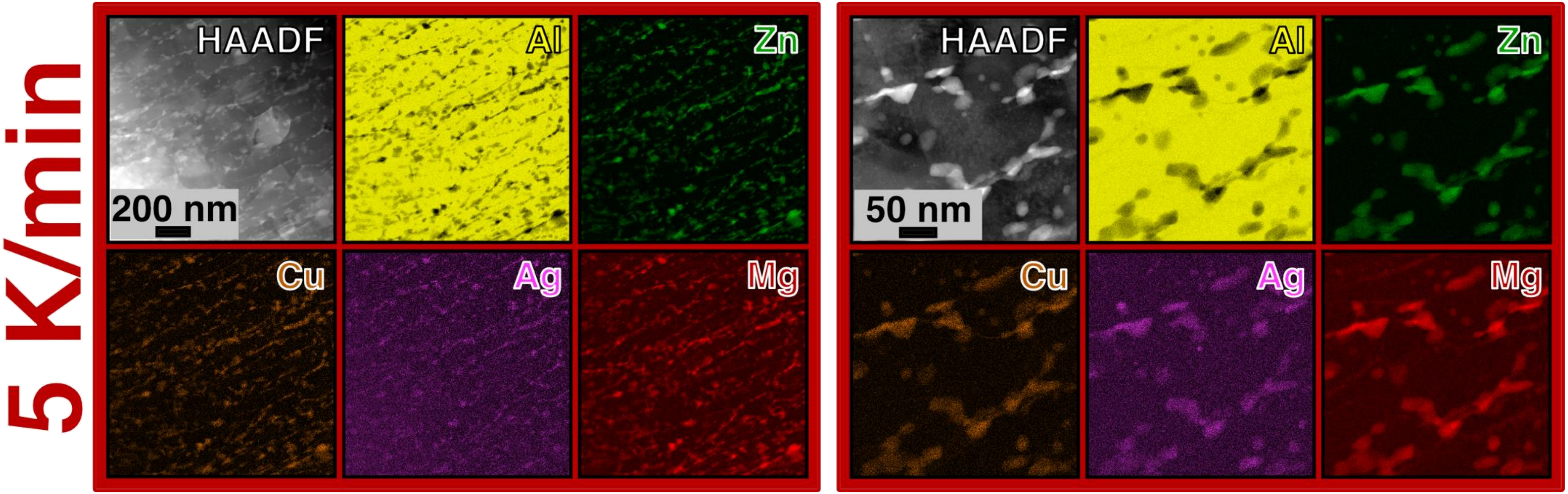

Supplemental Information 1 – Microstructure after annealing up to 506 K (STEM-EDX)

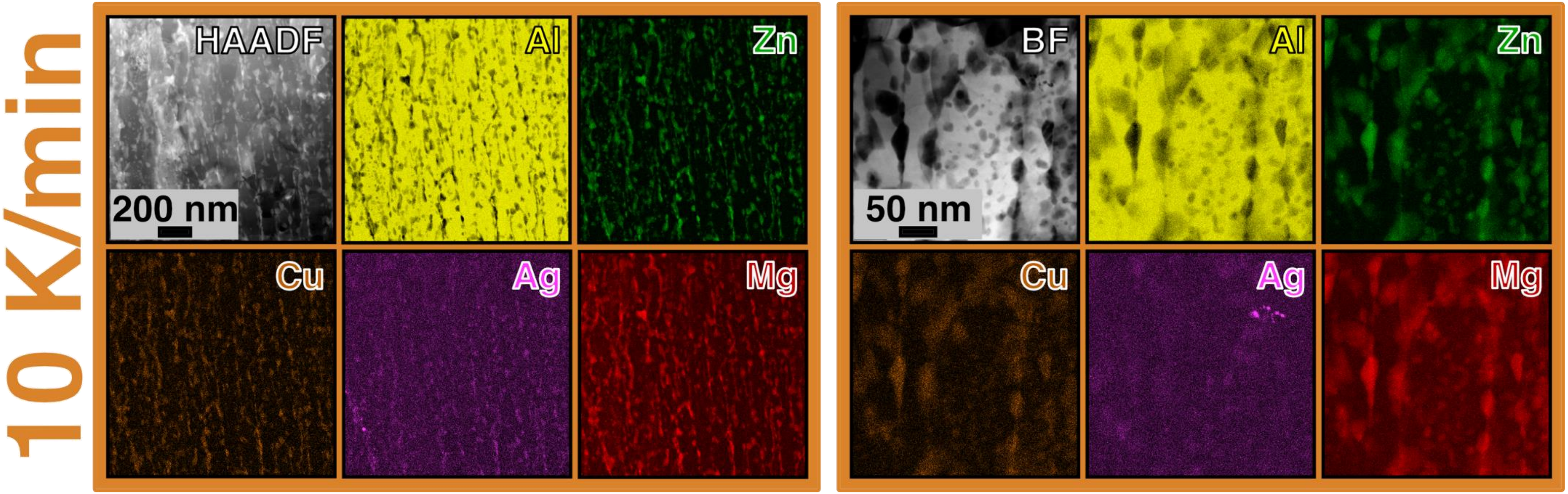

Supplemental Information 1 – Microstructure after annealing up to 506 K (STEM-EDX)

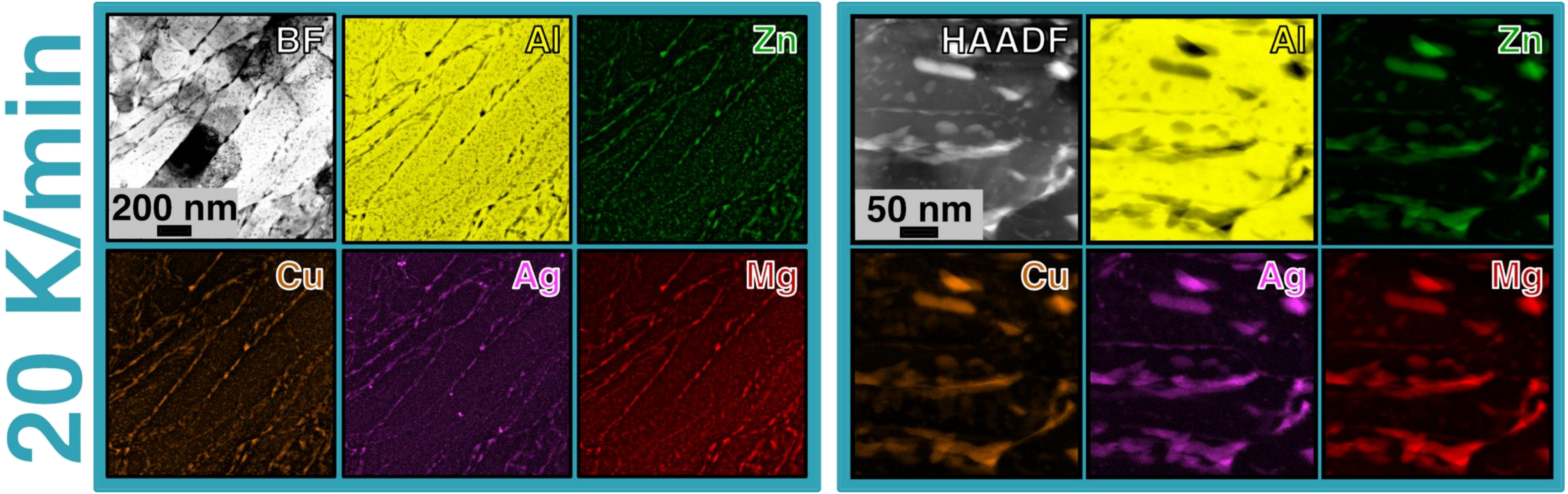

Supplemental Information 1 – Microstructure after annealing up to 506 K (STEM-EDX)

130 K/s

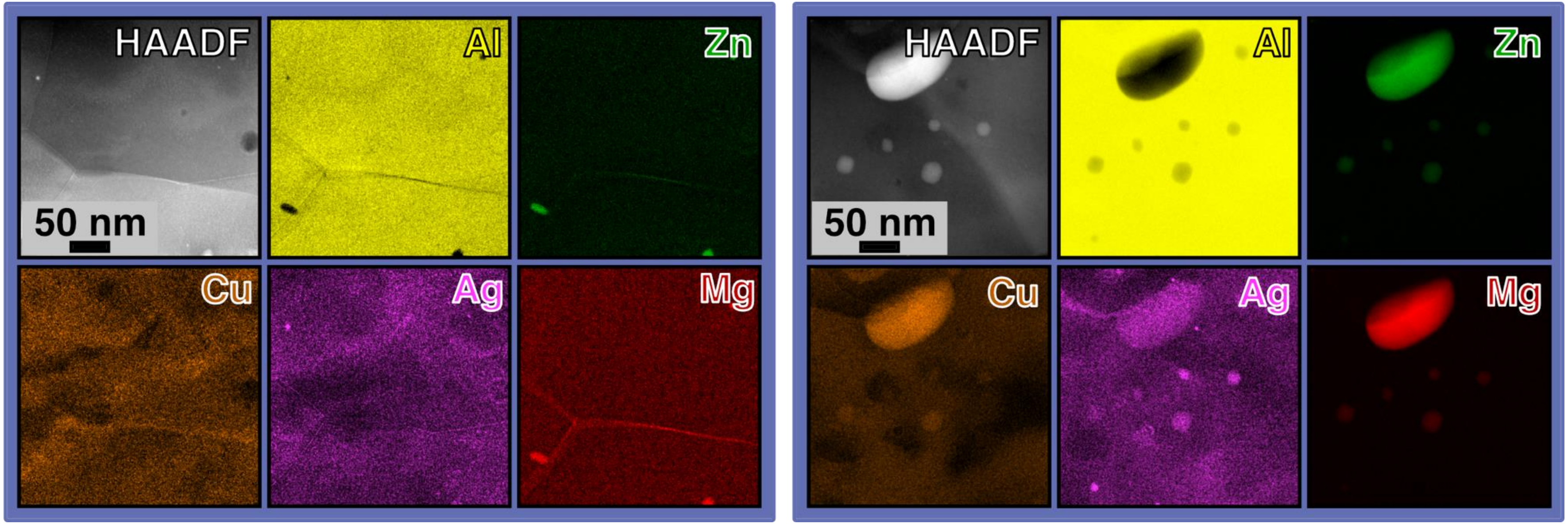

Supplemental Information 2 - In situ TEM annealing with the Gatan holder up to 506 K

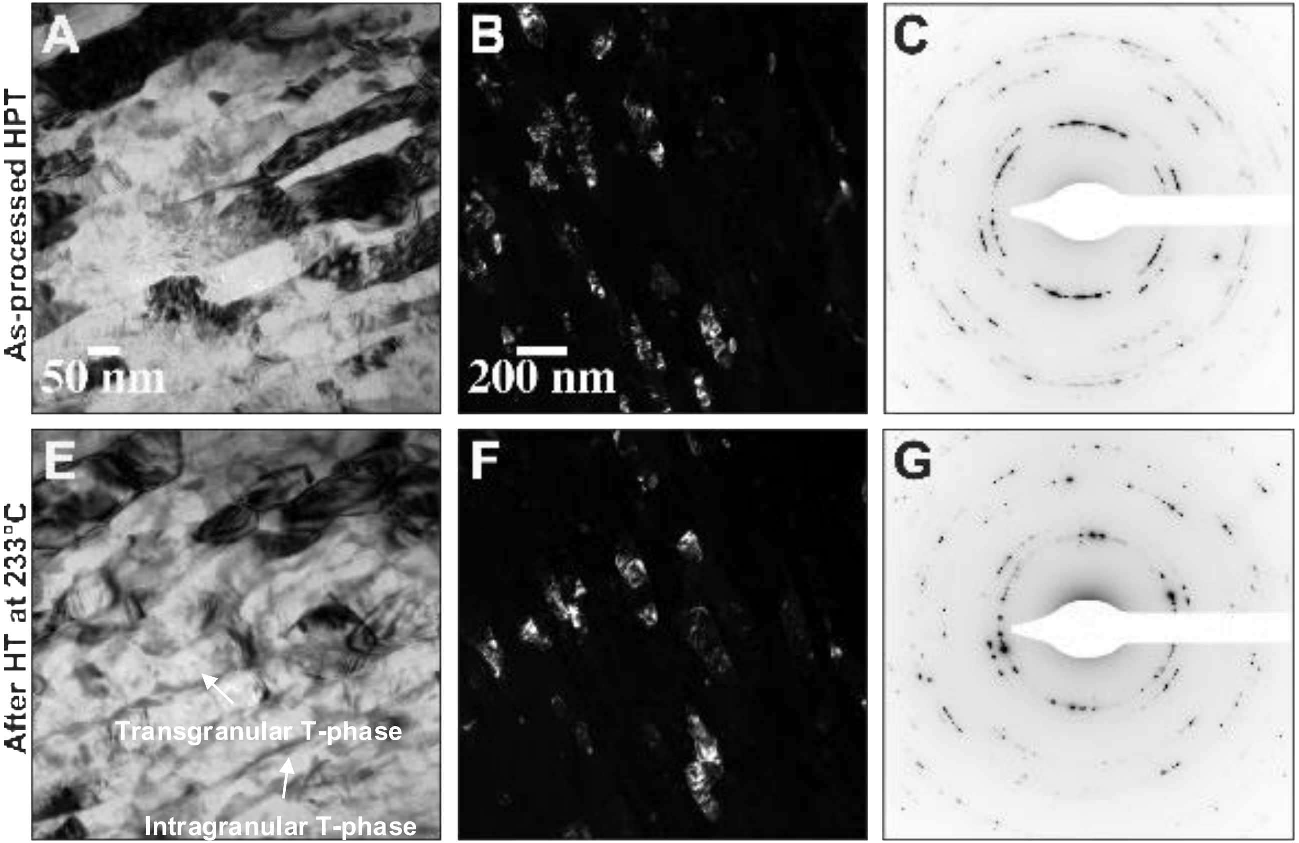

10 K/min heating rate

Supplementary Information 3 - *In situ* TEM ion irradiation

- Beam: 300 keV Ar ions (at 18.5º).
- Flux: $7.74 \cdot 10^{13}$ ions·cm^{-2}·s^{-1}.
- Dose: $2.30 \cdot 10^{17}$ ions·cm^{-2} (100 dpa over 100 nm thick samples).
- Using R.E. Stoller method, **red curves below** (https://doi.org/10.1016/j.nimb.2013.05.008).

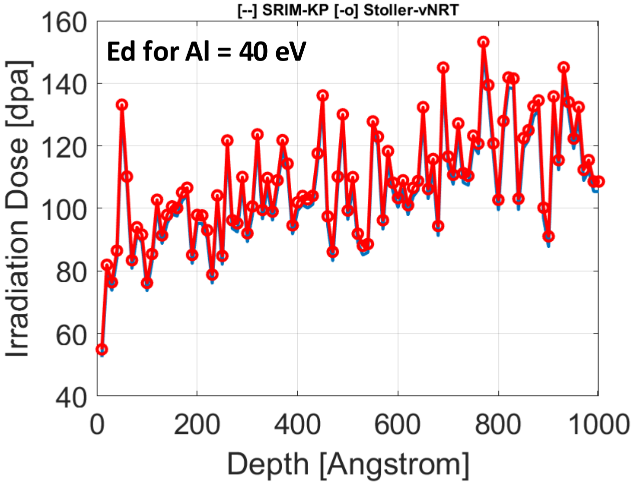

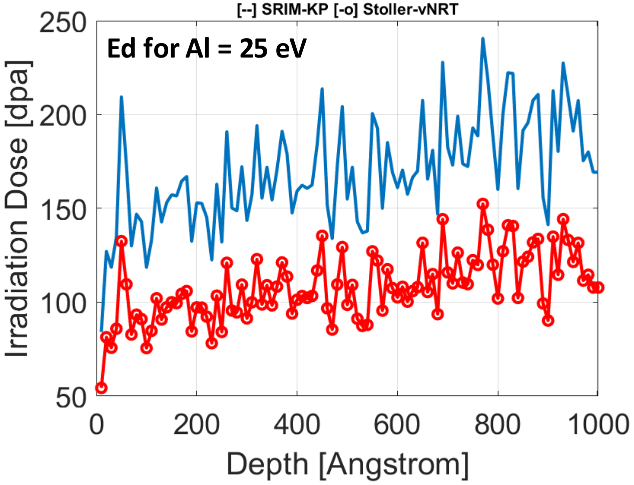

Supplementary Information 4 - *Ex situ* TEM ion irradiation for mechanical testing

- Beam: 600 keV Ne ions (at 18.5º).
- Each side of the specimens were irradiated. Calculations here show only one side.
- Flux: $4.40 \cdot 10^{12}$ ions$\cdot$cm$^{-2}\cdot$s^{-1}.
- Dose: $5.15 \cdot 10^{16}$ ions$\cdot$cm^{-2} (~ 13 dpa peak).
- Dose: $8.24 \cdot 10^{16}$ ions$\cdot$cm^{-2} (~ 20 dpa peak).

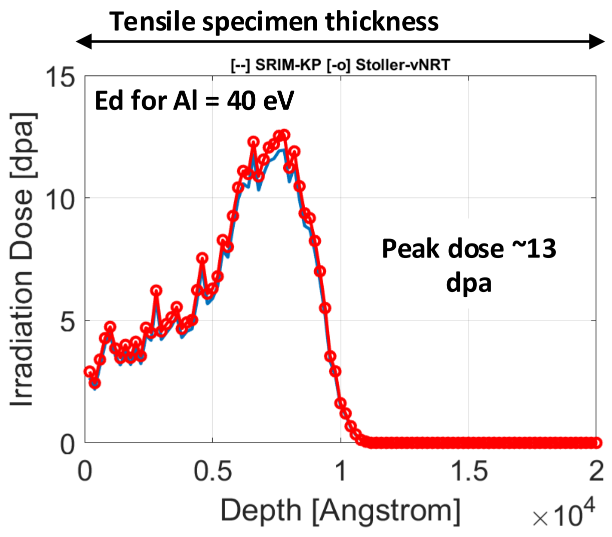

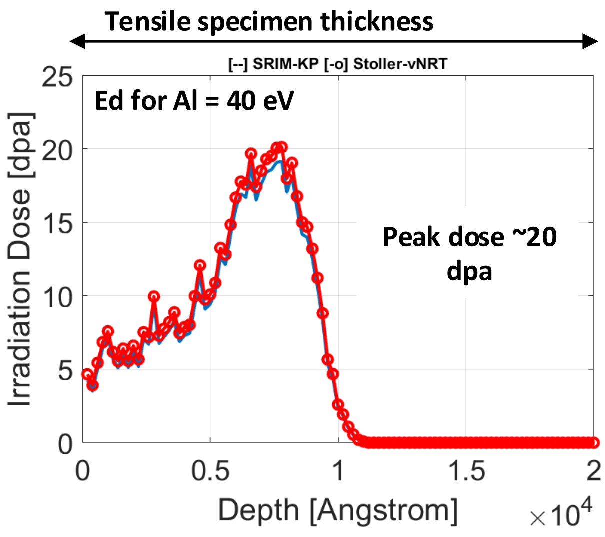

Supplementary Information 5 – FactSage Calculations

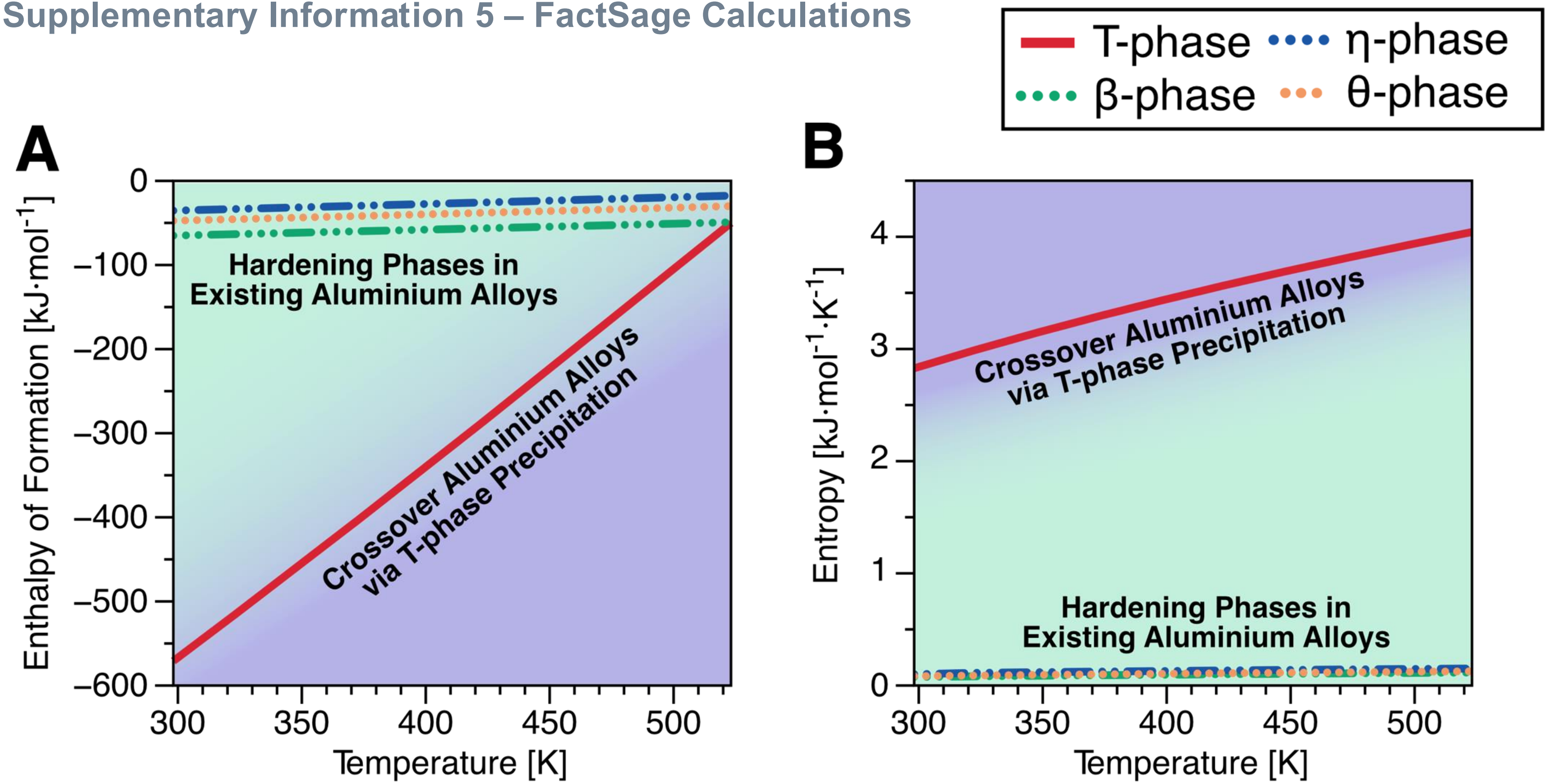

Both calculations express the Enthalpy and Entropy of the whole compounds
